# A seasonal investigation of indoor air quality in relation to architectural features in government office buildings in Enugu, Nigeria

**DOI:** 10.1038/s41598-024-78160-5

**Published:** 2024-11-06

**Authors:** Amaka-Anolue Martha Basil, Chiamaka Christiana Okwuosa, Francis Onyechi Uzuegbuanam, Lawrence E. Ugwu

**Affiliations:** 1 Department of Architecture, Faculty of Environmental Studies, University of Nigeria, Nsukka, Nigeria; 2 North-West University Mafikeng, Mahikeng, South Africa

**Keywords:** Architectural features, Concentration/level, Indoor air quality, Office building, Seasonal variations, Environmental sciences, Environmental social sciences

## Abstract

Indoor air quality (IAQ) is crucial to environmental health significantly impacting on the well-being and productivity of building occupants. Several studies have explored various aspects of IAQ in non-tropical regions but there is limited information on how seasonal variations affect IAQ in hot-humid climates like Enugu, Nigeria. This study investigated seasonal changes in key indoor air parameters including CO, CO_2_, HCHO, TVOC, temperature, RH, PM_2.5_, and AQI across rainy and dry seasons in 58 government offices in Enugu, and evaluated how these fluctuations relate to architectural features of the offices. Using a mixed-methods approach, data collection involved qualitative assessments of building design attributes alongside quantitative IAQ measurements taken with the BOSEAN T-Z01Pro detector. Seasonal variations were analyzed using paired T-tests, ANOVA, and regression models. The results revealed a marked increase in pollutant concentrations during the dry season (*p* < 0.001–0.005), resulting in a poorer air quality index compared to the rainy season. Architectural features accounted for 68.5% of the variability in AQI (R^2^ = 0.685, *p* = 0.000), with casement windows being significantly associated with better air quality (Exp B = -4.217, *p* = 0.013) These shows that the dry season poses a greater risk to IAQ which is worsened in offices where projecting windows were used, potentially impacting health and productivity. The study emphasizes the need to address seasonal IAQ differences when designing office buildings in tropical regions. Design architects can help reduce seasonal air quality challenges and support healthier, more productive indoor environments by incorporating ventilation strategies, such as the use of casement windows. Further research should include long-term monitoring across various settings and additional IAQ parameters for better understanding of IAQ dynamics.

**Supplementary Information:**

The online version contains supplementary material available at 10.1038/s41598-024-78160-5.

## Introduction

Indoor air quality (IAQ) is a critical aspect of environmental health, significantly influencing human well-being and productivity. According to the United States Environmental Protection Agency^1^, indoor pollutant levels often surpass outdoor levels by 2 to 5 times, and this emphasizes the severity of indoor air pollution^2^. The World Health Organization (WHO) ranks poor IAQ as the eighth most critical risk factor contributing to the global disease burden and accounting for approximately 2.7% of health impacts^3,4^. This highlights the urgent need for effective interventions to enhance IAQ, especially in office environments where individuals spend a substantial portion of their time.

Previous studies have highlighted the critical role of human behaviour and building characteristics in influencing indoor air quality (IAQ), especially in buildings occupied by vulnerable populations. Some of these emphasised the direct impact of occupant’s activities on fine particulate matter (PM2.5) levels, which pose serious health risks^5,6^. Both indoor and outdoor sources significantly contribute to indoor pollutant levels, as evidenced in review studies done in Boston^7^ and across European cities^8^. Another study aimed at assessing the occupant-behaviour-based indoor air quality further examined the impact of external environmental factors and how outdoor pollution infiltrates indoor spaces and exacerbates respiratory risks, particularly when window operation is factored in^9^.

Studies has shown that the integration of technological and design principles has a promising approach to improving IAQ as architectural design strategies involves deliberate approaches and techniques used to address specific challenges or achieve particular goals in building design. Building layout, construction materials, and ventilation methods significantly influence indoor pollutant levels^10–14^. Equipment and activities within office spaces further contribute to indoor pollution^15^. Recognizing these factors, research has explored various aspects of IAQ in non-tropical office buildings, focusing on indoor volatile organic compounds (VOCs), particulate matter, and the impact of HVAC systems on air quality^16–18^. It has been suggested that building design should undergo a shift, focusing on implementing ventilation systems that adjust to the specific needs of occupants^19^, considering that the continued emphasis on energy efficiency can sometimes overshadow concerns about indoor air quality, which may lead to negative impacts on occupant health^20^. Some studies have further explored the role of building materials, ventilation systems, and natural airflows, suggesting that advanced modelling and multi-data stream approaches could be effective in identifying and reducing indoor air quality hazards^8,21^. These studies show the need for holistic, strategies to enhance IAQ, modification, of building design, and frameworks to reduce the adverse health effects of indoor air pollution.

Furthermore, despite extensive research in non-tropical climates, limited knowledge exists about seasonal IAQ variations in office buildings within the hot-humid tropical climate of Enugu, Southeast Nigeria. Previous studies have primarily focused on the architectural designs of residential buildings and prisons, leaving a significant research gap in understanding IAQ in office environments where much of the public-sector workforce operates^3,22^. The architectural design attributes of government office buildings in Enugu are believed to significantly influence the seasonal variation of IAQ. Factors such as building orientation, construction materials, ventilation methods, and office equipment use are hypothesized to predispose these buildings to poor IAQ, especially given the unique geographical location, climatic conditions, and population-related environmental activities of the region.

Considering the distinct architectural features and climatic conditions of Enugu as well as the paucity of studies evaluating IAQ in Nigeria, this study aims to address the following objectives:^1^ to describe the architectural design attributes of selected office buildings in Enugu;^2^ to determine the seasonal concentration levels of major indoor air parameters (CO, CO_2_, HCHO, TVOC, Temperature, RH, PM_2.5_, AQI) in these office buildings across the rainy and dry seasons; and^3^ to investigate the relationship between the changes in the seasonal levels of IAQ parameters and the architectural features of the offices. By addressing these objectives, this study provides insights into the influence of architectural design on IAQ, thereby contributing to healthier working environments and enhanced productivity in government office buildings in Enugu, Nigeria.

## Materials and methods

**Fig. 1:**
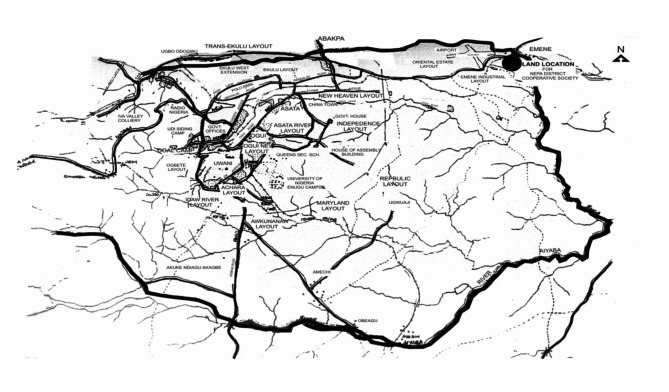
Map of Enugu metropolis^24^.

The study area is the Enugu metropolis, one of the major cities in the region, located in the state of south-eastern Nigeria (Fig. 1). It is located on the eastern periphery of Udi Cliff and lies between latitude 6.4610 N and longitude 7.4940E within the tropical hot-humid region of Nigeria. The discovery of coal mining in 1909 by engineer Mr. Kitson led to the establishment of a railway line from Port Harcourt through Enugu for coal export, attracting migrants and leading to planned construction for coal miners and civil servants. Enugu gained township status in 1917 under Lord Lugard’s Ordinance and has been the capital of Enugu State since its creation in 1991. Enugu’s topography is undulating with hills, and it is situated 200 m above sea level, part of the Nsukka-Udi-Okigwe Cuesta. The city experiences conventional and disturbance-related rainfall, with an annual mean ranging from 1500 mm to 1750 mm^22,23^.

The research focuses on the Enugu metropolis, which houses approximately 50 government parastatals, including 23 state government and 27 federal government parastatals. The study population includes offices from all three government tiers: Federal, State, and Local Government, all within the metropolis. Each secretariat building has unique features but shares similarities within its category.

The research design utilised in this study involved a case study approach, focusing on multiple sites and incorporating both experimental and survey research designs. This decision was driven by the necessity to comprehensively ascertain the concentration/level of the main indoor air quality (IAQ) parameters within office buildings during rainy and dry seasons (the two major seasons in the region). Additionally, it aimed to enhance understanding regarding the relationship between the architectural characteristics of office buildings and the seasonal variations in IAQ parameters. Furthermore, the study sought to explore how architectural features may impact seasonal IAQ in office buildings in the hot and humid tropics of Enugu, southeast Nigeria.

Specific criteria were established to determine the sample size of the offices by examining various office types/ layouts, including private offices, open-plan offices, and traditional/ cubicles office. Factors such as their cardinal directions (North, South, East, or West) and their position within the office complex (ground floor or suspended floor) were also considered. These criteria allowed for classifying office types into distinct models, each sharing similarities with others in its category. Furthermore, additional selection determinants included office surface area, and counts of furniture, headroom (floor to ceiling height), window characteristics (types, sizes, and numbers), occupant numbers, floor/wall/ceiling finishes, surrounding landscapes, presence of electronic devices, and other architectural features.

Following these selection guidelines, a sample size of 58 offices was identified for the study. This comprised 24 offices from the Federal Government secretariat complex, 24 from the Enugu State Government secretariat complex, and 10 from the Enugu North Local Government secretariat complex. This approach ensured a well-balanced representation of various office buildings with varying characteristics within the designated research area.

The BOSEAN Multi-function office/home air Quality detector, model T-Z01Pro, an 8-in-1 Air Quality Detector^25^. It was employed to assess the office Indoor Air Quality parameters of interest. Chosen for its comprehensive capabilities, this device has four sensors that concurrently measure PM2.5, HCHO, TVOC, CO, and CO_2_ concentrations in the air, along with detecting temperature, humidity, and the Air Quality Index (AQI). The detector offers precise measurement accuracy and ranges of 0–1.999 mg/m3 for HCHO, 0–9.999 mg/m3 for TVOC, and 0–999 µg/m3 for PM2.5, ensuring both efficiency and accuracy.

Also, a carefully prepared observation schedule was developed to facilitate the comprehensive field survey and capture detailed information regarding all investigated office buildings’ architectural features, occupancy, furnishing details, and surrounding attributes. Each office was systematically coded on the observation schedule in relation to the office complex design floor plans to ensure ease of identification and location. The coding system employed a combination of cardinal point orientation (A for north, B for east, C for west, and D for south), office layout type (1 for private office, 2 for open plan office, and 3 for traditional/ cubicle office), and location within the building complex (1 for ground floor and 2 for suspended floor), with layout and location denoted as subscripts for clarity and precision.

The study employed a combination of qualitative and quantitative research approaches. For the qualitative research, a well-articulated observation schedule was used to identify the architectural design features of the selected office buildings. For quantitative analysis, which constitutes an experimental approach, the BOSEAN Multi-function office/home air quality detector, model T-Z01Pro, an 8-in-1 Air Detector, was employed to evaluate the concentration/level of the Indoor Air Quality parameters under investigation. The instrument was calibrated using manufacturer’s recommended procedures on the operation manual. This involved placing the machine in clear air, running it for 15 min with an unobstructed air vent, and selecting “set” on the device to initiate automatic calibration^25^. To ensure precise measurements, the detector was strategically positioned at the centre of the office floor, away from aisles, to minimize disruption from daily office activities. To replicate the breathing zone of a seated office worker, the monitor was positioned 1 m above the floor on a desk. Data were collected at 15-minute intervals in real-time, commencing from 8 am (the start of the workday) to 4 pm (the end of the workday), and their averages were calculated. The data collection period spanned four consecutive days per office, from Tuesday to Friday, both in the rainy (May to August) and dry seasons (November to December). Measurements were taken in 57 offices out of the sample size of 58 offices.

The experimental design aimed for the field survey to encompass a range of indoor climatic conditions, stretching across the two major seasons in Nigeria (Rainy and Dry Seasons). The survey’s structure was calculatedly crafted to facilitate daily record-keeping, fostering a precise and comprehensive assessment of the indoor air quality of the case study office buildings. To ensure thoroughness, each office was surveyed on at least four occasions per season.

The Statistical Program for Social Sciences 23 was used to evaluate measurement data statistically. Mean and standard deviation values were calculated using this program. Additionally, the paired T-test was used to determine the seasonal variations and significance of the IAQ parameters. Finally, analysis of variance (ANOVA) was used to determine the statistically significant relationship between the variables and the architectural features of the three different study locations as it relates to seasonal changes.

## Result

### Architectural design attributes of selected office buildings

The architectural design features of the selected government office buildings in the Enugu Metropolis, Nigeria, are shown in Table 1. Among the examined office buildings, 21.1% had a floor area ranging from 10 m^2^ to 19 m^2^, 29.9% fell in the 20 m^2^ to 29 m^2^ range, and 28.1% spanned from 40 m^2^ to 49 m^2^. Collectively, these three categories constituted 79.1% of the total offices investigated. Location 1 had the most offices with larger floor areas (above 50 m^2^).

Regarding headroom, 61.4% of the offices in the study area exceeded 3 m, while 38.4% had headroom between 2 m and 3 m. Most (74.8%) of the studied offices had volumes more significant than 50 m^3^, with only 7% having volumes below 50 m^3^. The predominant window type observed in the offices was Projecting windows, with 57.9% of the window sizes falling within the range of 1.2 m x 1.2 m to 1.2 m x 1.5 m, while the remaining 42.1% were smaller than 1.2 m x 1.2 m. There were no other openings apart from the primary windows and doors in the office spaces.

All the offices have overhangs and eaves as external shading devices. In terms of office form and layout, Circular and H-shaped forms were the predominant building forms, accounting for 42.1% and 40.4%, respectively, while private, open-plan, and traditional/cubicle office layouts were the primary office layouts/types in the study area, each accounting for about a third of the total number of offices.

Among the offices studied, 87.7% have ceramic floor tiles, while the walls are painted (42.1%) or wood-finished (57.9%). The ceilings are mostly concrete slabs finished with paint (57.9%) and asbestos finished with paint (42.1%). Only 29.8% of the offices studied are partitioned with wood, while about two-thirds have no partitions.

Regarding orientation, about 57.9% of the office complexes had longer sides facing East-West, while 42.1% had longer sides-oriented North-South. Approximately 57.9% of the office surroundings had soft and hard landscaping elements (see Table 1).

**Table 1:** Architectural design attributes of the selected office buildings.

S/n	Architectural design attributes		Location I	Location II	Location III	Frequency *N*(%)
1	Office floor area	Below 10 m^2^	-	-	2	2(3.5)
		10 m^2^–19 m^2^	-	7	5	12(21.1)
		20 m^2^–29 m^2^	8	8	1	17(29.8)
		30 m^2^–39 m^2^	-	-	2	2(3.5)
		40 m^2^–49 m^2^	8	8	-	16(28.1)
		50 m^2^ and above	8	-	-	8(14.0)
2	Office headroom	2–3 m	-	12	10	22(38.6)
		Above 3 m	24	11	-	35(61.4)
3	Office volume	Below 50 m^3^	-	-	4	4(7.0)
		50 m^3^ – 100 m^3^	8	7	4	19(33.3)
		101 m^3^- 150 m^3^	8	8	2	18(31.6)
		Above 150 m^3^	8	8	-	16(43.2)
4	Types of windows in offices	Projecting alone	24	-	-	24(42.1)
		Projecting and louvers	-	23	-	23(40.4)
		Casement alone	-	-	10	10(17.5)
5	External shading devices	Overhangs and eaves	24	23	10	57(100.0)
6	Building form	Circular	24	-	-	24(42.1)
		H-shaped	-	23	-	23(40.4)
		Rectangular	-	-	10	10(17.5)
7	Office layout/type	Private office	8	7	4	19(33.3)
		Open plan office	8	8	4	20(35.1)
		Cubicle/Traditional office	8	8	2	18(31.6)
8	Number of windows in office	1	-	-	2	2(3.5)
		2	-	-	3	3(5.3)
		3	-	9	1	10(17.5)
		Above 3	24	14	4	42(73.7)
9	Sizes of office windows	Below 1.2 m × 1.2 m	24	-	-	24(42.1)
		1.2 m × 1.2 m- 1.2 m × 1.5 m	-	23	10	33(57.9)
10	Office floor finish	Ceramic tile	24	21	5	50(87.7)
		Rug	-	2	1	3(5.3)
		Terrazzo	-	-	4	4(7.0)
11	Office wall finish	Paint	24	-	-	24(42.1)
		Wood and paint	-	23	10	33(57.9)
12	Office ceiling finish	Asbestos and paint	24			24(42.1)
		Concrete slab and paint		23	10	33(57.9)
13	Office partitioning material	None	16	15	8	39(68.4)
		Wood	8	8	1	17(29.8)
		Wood and glass			1	1(1.8)
14	Other office openings	None	24	23	10	57(100.0)
15	Office building orientation	Longer part facing North-South	24	-	-	24(42.1)
		Longer part facing East-West	-	23	10	33(57.9)
16	Surrounding landscape	Paved with Asphalt/ concrete	24	-	-	24(42.1)
		Landscaped with plants	-	23	-	23(40.4)
		Paved with Coal tar and little plants	-	-	10	10(17.5)

### Seasonal concentration/level of the IAQ parameters in the selected office buildings across the rainy and dry seasons

There was a significant difference in the mean of most IAQ parameters (CO_2_, HCHO, TVOC, temperature, RH and PM2.5; p-value < 0.001–0.005) taken from the studied buildings across the rainy and dry seasons with the exception of CO. The overall AQI was significantly lower in the rainy season compared to the dry season (p-value < 0.001). Higher levels of CO_2_ and PM2.5 were particularly noted to be significantly higher in the dry season (see Table 2). Detailed concentration of the investigated IAQs in the selected office buildings across the rainy and dry seasons are shown in the tables attached (Suppl. 1).

**Table 2:** Comparison of IAQ parameters across the rainy and dry seasons.

S/no	IAQ parameters	Mean	Std. deviation	95% confidence interval		*p*-value
				Lower	Upper	
1	Rainy Season Average Carbon(II)Oxide (CO) Concentration - Dry Season Average Carbon(II)Oxide (CO) Concentration	0.105	0.838	− 0.117	0.328	0.347
2	Rainy Season Average Carbon(IV)Oxide (CO_2_) Concentration - Dry Season Average Carbon(IV)Oxide (CO_2_) Concentration	12.000	24.340	5.542	18.458	0.000*
3	Rainy Season Average Fomaldehyde (HCHO) Concentration - Dry Season Average Fomaldehyde (HCHO) Concentration	0.014789	0.011063	0.011854	0.017725	0.000*
4	Rainy Season Average Total Volatile Organic Compound (TVOC) Concentration - Dry Season Average Total Volatile Organic Compound (TVOC) Concentration	0.028298	0.072195	0.009142	0.047454	0.005*
5	Rainy Saoson Average Office Indoor Temperature - Dry Saoson Average Office Indoor Temperature	-1.123	1.763	-1.591	− 0.655	0.000*
6	Rainy Season Average Relative Humidity (RH) - Dry Season Average Relative Humidity (RH)	6.789	4.246	5.663	7.916	0.000*
7	Rainy Season Average Particulate Matter 2.5Microns (PM2.5) Concentration - Dry Season Average Particulate Matter 2.5Microns (PM2.5) Concentration	-6.474	4.355	-7.629	-5.318	0.000*
8	Rainy Season Office Air Quality Index (AQI) - Dry Season Office Air Quality Index (AQI)	-2.018	4.151	-3.119	− 0.916	0.001*

### Relationship between the changes in the seasonal IAQ and the architectural features of the offices

The multiple linear regression model explained approximately 68.5% of the variance in AQI changes (R^2^ = 0.685, Adjusted R^2^ = 0.624), indicating a strong relationship between the architectural features and AQI changes. The regression model was statistically significant (*p* = 0.000), suggesting that the architectural features collectively have a significant effect on AQI changes.

The types of windows in the offices had a significant negative impact on AQI changes (Exp B = -4.217, *p* = 0.013), indicating that casement windows can significantly reduce AQI levels compared to other window types. Offices with casement windows had the lowest pollutant concentrations, while those with projecting windows had the highest. Other architectural features included in the model did not show a statistically significant relationship with AQI changes individually (see Table 3).

**Table 3:** Multiple linear regression analysis of architectural parameters on AQI changes.

Architectural features	Exp B	95% CI Exp B		SE	t-value	*p*-value
		Lower	Upper			
Constant	*− 0.389*	*-13.567*	*12.790*		*− 0.059*	*0.953*
Office layout	0.222	-2.577	3.022	6.551	0.160	0.874
Office headroom	0.205	-1.986	2.397	1.391	0.189	0.851
Office floor area	-1.062	-5.058	2.934	1.089	− 0.535	0.595
Office volume	1.121	-4.306	6.547	1.986	0.416	0.680
Number of windows in offices	1.218	− 0.978	3.414	2.697	1.116	0.270
Types of windows in offices	-4.217	-7.485	− 0.949	1.092	-2.596	0.013
Office floor finish	0.872	-1.853	3.598	1.625	0.644	0.523
Office partitioning materials	− 0.173	-3.469	3.123	1.355	− 0.106	0.916
Surrounding landscape	0.442	-1.360	2.244	1.639	0.493	0.624
Model summary	ANOVA					
R-squared: 0.685	F-statistic: 11.33					
Adjusted R-squared: 0.624	p-value: 0.000					

Significant values are in [italics].

## Discussion

The evaluation of government office buildings in the Enugu Metropolis, Nigeria, reveals several architectural design features affecting indoor air quality (IAQ). Most offices have headroom of 3 m or less, falling below the ASHRAE 55.0 comfort limit, which may impede IAQ and compromise the health and productivity of office users in Nigeria’s hot, humid climate^26^. Additionally, the study found that most offices were solely designed with projecting windows, restricting airflow and significantly affecting IAQ^22,27,28^. Wooden partitions may further degrade IAQ due to their hygroscopic properties^29^. Recommendations include using alternative partitioning materials and window types to enhance airflow and IAQ. Building morphology, particularly circular and H-shaped designs, influences IAQ by shaping outdoor air quality, with a notable link between outdoor and indoor air quality^30,31^. Furthermore, the prevalent East-West orientation of buildings impacts IAQ^32^, while the scarcity of green landscapes undermines the potential benefits of greenery on IAQ^10,33^. These insights highlight the need for design architects to consider these factors to improve IAQ, comfort, and productivity in office environments in the hot humid sub-African region.

The findings in this study align with previous studies that have assessed seasonal variations in IAQ. Some of which found that IAQ parameters often fluctuate with seasonal changes, largely due to variations in ventilation rates, temperature, and humidity while others reported that pollutant concentrations such as CO_2_ and PM2.5 tend to be higher during the dry season due to increased outdoor pollutant levels as observed in the index study^34–37^. This implies that poor IAQ, particularly during the dry season, can lead to a range of health issues such as respiratory problems, headaches, fatigue and mental exhaustion, which can significantly compromise mental health, reduce productivity and increase absenteeism. Also, prolonged exposure to high levels of pollutants like CO_2_ and PM2.5 can have severe long-term health impacts, including chronic respiratory diseases and cardiovascular issues^4,38^.

The study highlights a significant relationship between changes in seasonal IAQ and the architectural features of office buildings in the study area. Notably, the types of windows in offices had a significant negative impact on AQI changes, emphasizing the role of window design in mitigating indoor air pollutants. This aligns with previous studies that have highlighted the importance of natural ventilation and window design in improving indoor air quality^22,27^. This finding emphasizes the need for prioritizing use of casement windows over projecting windows in office spaces to enhance the working environment and overall well-being of the office users. It also highlights the need for architects to critically consider the chosen window types in office buildings so as to optimize air quality, particularly in the hot-humid tropical climates like Enugu. By incorporating effective window designs, architects can significantly improve indoor air quality, thereby promoting healthier and more productive office environments.

This study has some limitations that necessitate further research. Firstly, the study is confined to a specific region and building type, which may not reflect IAQ conditions elsewhere. As such, future research should include a broader range of geographical locations and different building types, such as commercial, residential, and educational structures, to improve the generalizability of the results. Secondly, the study only captures IAQ parameters during specific periods in the rainy and dry seasons. Short-term monitoring may not adequately reflect long-term IAQ trends or fully capture seasonal variations. Extending the monitoring period to cover multiple seasons and years would provide a more comprehensive understanding of IAQ dynamics. Thirdly, the study’s reliance on a limited set of IAQ parameters, such as CO_2_, HCHO, TVOC, temperature, RH, and PM2.5, excludes other important factors like biological contaminants and a wider range of VOCs. Therefore, including a broader array of IAQ parameters in future studies would offer a more holistic view.

## Conclusion

The study aimed at assessing the seasonal concentration of indoor air quality (IAQ) parameters in government office buildings in Enugu, Nigeria, and investigating their relationship with architectural design features. It showed strong relationship between these features and AQI changes, particularly highlighting the types of windows as a critical factor in reducing indoor air pollutants. These findings suggest that poor IAQ, particularly in the dry season, can adversely affect the physical and mental health, and productivity of office users. Future research should broaden the scope to include diverse geographical locations and building types, extend monitoring periods to capture long-term IAQ trends, and consider a wider range of IAQ parameters to provide a more comprehensive understanding of IAQ dynamics.

## Electronic supplementary material

Below is the link to the electronic supplementary material.

Supplementary Material 1

## Data Availability

Data would be made available by the corresponding author upon request.
