# Supplementary material for: A seasonal investigation of indoor air quality in relation to architectural features in government office buildings in Enugu, Nigeria
Source: Sci Rep. 2024 Nov 6;14:26885. doi: 10.1038/s41598-024-78160-5 (PMC11541727; doi:10.1038/s41598-024-78160-5)
Supplement: Supplementary file 1 — Supplementary Material 1 [file 41598_2024_78160_MOESM1_ESM.docx]

**Table S1: Concentration of the Main Indoor Air Parameters in The Selected Office Buildings in the Study Area I**

**Office Location: I**

**Season: Wet/ Rainy Season**

**Inspection Date: May – August, 2023**

| **Cardinal point Location** | **Office Layout Type** | | **Rainy Season Average Carbon(II)Oxide (CO) Concentration (ppm)** | **Rainy Season Average Carbon(IV)Oxide (CO2) Concentration (ppm)** | **Rainy Season Average Formaldehyde (HCHO) Concentration (mg/m^3^)** | **Rainy Season Average Total Volatile Organic Compound (TVOC) Concentration (mg/m^3^)** | **Rainy Season Average Office Indoor Temperature (^0^C)** | **Rainy Season Average Relative Humidity (RH) (%)** | **Rainy Season Average PM 2.5Microns (PM2.5) Concentration (ug/m^3^)** | **Rainy Season Office Air Quality Index (AQI)** |
| --- | --- | --- | --- | --- | --- | --- | --- | --- | --- | --- |
| **North** | **Private**  **((N= 2))** | **Mean** | 3.00 | 410.50 | .02150 | .05850 | 26.00 | 70.50 | 6.50 | 6.00 |
|  |  | **SD** | .000 | 6.364 | .000707 | .003536 | .000 | .707 | .707 | 1.414 |
|  | **Open Plan**  **(N= 2)** | **Mean** | 3.00 | 420.00 | .02450 | .07550 | 26.00 | 71.00 | 7.00 | 6.00 |
|  |  | **SD** | .000 | 7.071 | .000707 | .002121 | .000 | 1.414 | 1.414 | 1.414 |
|  | **Cubicle /Traditional (N= 2)** | **Mean** | 2.50 | 421.50 | .01650 | .04250 | 26.00 | 72.00 | 6.50 | 6.50 |
|  |  | **SD** | .707 | 4.950 | .009192 | .045962 | .000 | 1.414 | .707 | .707 |
|  | **Total**  **(N= 6)** | **Mean** | 2.83 | 417.33 | .02083 | .05883 | 26.00 | 71.17 | 6.67 | 6.17 |
|  |  | **SD** | .408 | 7.174 | .005492 | .025373 | .000 | 1.169 | .816 | .983 |
| **East** | **Private**  **(N= 2)** | **Mean** | 2.50 | 414.50 | .03300 | .05400 | 26.00 | 71.50 | 5.50 | 6.00 |
|  |  | **SD** | .707 | .707 | .004243 | .045255 | .000 | .707 | .707 | 2.828 |
|  | **Open Plan (N= 2)** | **Mean** | 3.00 | 440.00 | .03200 | .11950 | 26.50 | 71.00 | 7.00 | 8.50 |
|  |  | **SD** | .000 | 2.828 | .005657 | .009192 | .707 | 1.414 | 1.414 | .707 |
|  | **Cubicle /Traditional (N= 2)** | **Mean** | 3.00 | 440.50 | .02350 | .09800 | 26.50 | 71.00 | 7.00 | 8.50 |
|  |  | **SD** | .000 | 3.536 | .013435 | .043841 | .707 | 1.414 | 1.414 | .707 |
|  | **Total**  **(N= 6)** | **Mean** | 2.83 | 431.67 | .02950 | .09050 | 26.33 | 71.17 | 6.50 | 7.67 |
|  |  | **SD** | .408 | 13.456 | .008240 | .041264 | .516 | .983 | 1.225 | 1.862 |
| **West** | **Private**  **(N= 2)** | **Mean** | 3.00 | 410.50 | .03200 | .03650 | 26.00 | 70.00 | 6.00 | 9.50 |
|  |  | **SD** | .000 | .707 | .009899 | .013435 | .000 | .000 | 1.414 | 4.950 |
|  | **Open Plan (N= 2)** | **Mean** | 4.00 | 462.50 | .03700 | .15350 | 26.50 | 68.00 | 8.50 | 11.00 |
|  |  | **SD** | 1.414 | 24.749 | .004243 | .075660 | .707 | .000 | 2.121 | 2.828 |
|  | **Cubicle /Traditional (N= 2)** | **Mean** | 3.50 | 458.50 | .03000 | .14900 | 26.50 | 68.00 | 8.50 | 7.50 |
|  |  | **SD** | 2.121 | 26.163 | .008485 | .079196 | .707 | .000 | 2.121 | 2.121 |
|  | **Total**  **(N= 6)** | **Mean** | 3.50 | 443.83 | .03300 | .11300 | 26.33 | 68.67 | 7.67 | 9.33 |
|  |  | **SD** | 1.225 | 30.486 | .006928 | .077141 | .516 | 1.033 | 1.966 | 3.141 |
| **South** | **Private**  **(N= 2)** | **Mean** | 4.00 | 470.00 | .05450 | .23300 | 25.50 | 70.00 | 6.00 | 11.50 |
|  |  | **SD** | 2.828 | 86.267 | .030406 | .288500 | .707 | .000 | 1.414 | 7.778 |
|  | **Open Plan (N= 2)** | **Mean** | 2.50 | 427.00 | .02800 | .07950 | 26.00 | 69.50 | 7.00 | 8.50 |
|  |  | **SD** | .707 | 25.456 | .009899 | .084146 | .000 | .707 | .000 | 3.536 |
|  | **Cubicle /Traditional (N= 2)** | **Mean** | 2.50 | 428.50 | .02950 | .06750 | 26.00 | 69.50 | 7.00 | 8.50 |
|  |  | **SD** | .707 | 26.163 | .009192 | .065761 | .000 | .707 | .000 | 3.536 |
|  | **Total**  **(N= 6)** | **Mean** | 3.00 | 441.83 | .03733 | .12667 | 25.83 | 69.67 | 6.67 | 9.50 |
|  |  | **SD** | 1.549 | 47.237 | .019967 | .160438 | .408 | .516 | .816 | 4.416 |

**Office Location: I**

**Season: Dry Season**

**Inspection Date: November – December, 2023**

| **Cardinal point Location** | **Office Layout Type** | | **Dry Season Average Carbon(II)Oxide (CO) Concentration (ppm)** | **Dry Season Average Carbon(IV)Oxide (CO2) Concentration (ppm)** | **Dry Season Average Formaldehyde (HCHO) Concentration (mg/m3)** | **Dry Season Average Total Volatile Organic Compound (TVOC) Concentration (mg/m3)** | **Dry Season Average Office Indoor Temperature (0C)** | **Dry Season Average Relative Humidity (RH) (%)** | **Dry Season Average PM2.5Microns (PM2.5) Concentration (ug/m3)** | **Dry Season Office Air Quality Index (AQI)** |
| --- | --- | --- | --- | --- | --- | --- | --- | --- | --- | --- |
| **North** | **Private**  **(N= 2)** | **Mean** | 3.00 | 406.50 | .02650 | .01300 | 26.00 | 65.00 | 10.00 | 6.00 |
|  |  | **SD** | .000 | 2.121 | .002121 | .000000 | .000 | 7.071 | .000 | .000 |
|  | **Open Plan**  **(N= 2)** | **Mean** | 3.00 | 404.50 | .01350 | .01150 | 26.50 | 62.50 | 12.50 | 8.00 |
|  |  | **SD** | .000 | 2.121 | .002121 | .002121 | .707 | 4.950 | .707 | .000 |
|  | **Cubicle /Traditional (N= 2)** | **Mean** | 3.00 | 408.00 | .01400 | .01550 | 26.00 | 64.00 | 11.50 | 7.50 |
|  |  | **SD** | .000 | 2.828 | .002828 | .006364 | .000 | 4.243 | .707 | .707 |
|  | **Total**  **(N= 6)** | **Mean** | 3.00 | 406.33 | .01800 | .01333 | 26.17 | 63.83 | 11.33 | 7.17 |
|  |  | **SD** | .000 | 2.422 | .006841 | .003502 | .408 | 4.446 | 1.211 | .983 |
| **East** | **Private**  **(N= 2)** | **Mean** | 3.00 | 410.00 | .01000 | .04250 | 26.50 | 64.00 | 9.50 | 7.00 |
|  |  | **SD** | .000 | 1.414 | .000000 | .010607 | .707 | 7.071 | 3.536 | 1.414 |
|  | **Open Plan**  **(N= 2)** | **Mean** | 3.00 | 409.00 | .01000 | .01850 | 26.50 | 63.00 | 9.50 | 7.00 |
|  |  | **SD** | .000 | 1.414 | .000000 | .017678 | .707 | 7.071 | 3.536 | 1.414 |
|  | **Cubicle /Traditional (N= 2)** | **Mean** | 3.00 | 408.50 | .01000 | .02250 | 27.00 | 69.00 | 8.50 | 6.00 |
|  |  | **SD** | .000 | .707 | .000000 | .017678 | .000 | .000 | 2.121 | .000 |
|  | **Total**  **(N= 6)** | **Mean** | 3.00 | 409.17 | .01000 | .02783 | 26.67 | 65.33 | 9.17 | 6.67 |
|  |  | **SD** | .000 | 1.169 | .000000 | .016726 | .516 | 5.317 | 2.483 | 1.033 |
| **West** | **Private**  **(N= 2)** | **Mean** | 3.00 | 413.00 | .01550 | .04250 | 27.00 | 65.00 | 9.50 | 6.50 |
|  |  | **SD** | .000 | 4.243 | .000707 | .003536 | .000 | .000 | .707 | .707 |
|  | **Open Plan**  **(N= 2)** | **Mean** | 3.00 | 415.00 | .01100 | .05250 | 27.00 | 66.50 | 10.00 | 6.50 |
|  |  | **SD** | .000 | 7.071 | .001414 | .017678 | .000 | 2.121 | 1.414 | .707 |
|  | **Cubicle /Traditional (N= 2)** | **Mean** | 3.00 | 415.00 | .01150 | .04500 | 27.00 | 68.00 | 9.50 | 6.50 |
|  |  | **SD** | .000 | 1.414 | .002121 | .021213 | .000 | .000 | .707 | .707 |
|  | **Total**  **(N= 6)** | **Mean** | 3.00 | 414.33 | .01267 | .04667 | 27.00 | 66.50 | 9.67 | 6.50 |
|  |  | **SD** | .000 | 3.882 | .002503 | .013292 | .000 | 1.643 | .816 | .548 |
| **South** | **Private**  **(N= 2)** | **Mean** | 2.50 | 400.50 | .01100 | .01050 | 24.00 | 53.50 | 8.00 | 5.50 |
|  |  | **SD** | .707 | .707 | .001414 | .000707 | 1.414 | 3.536 | 2.828 | 2.121 |
|  | **Open Plan**  **(N= 2)** | **Mean** | 2.50 | 405.50 | .01050 | .02950 | 23.00 | 60.50 | 9.00 | 6.50 |
|  |  | **SD** | .707 | 7.778 | .000707 | .028991 | .000 | 6.364 | 2.828 | .707 |
|  | **Cubicle /Traditional (N= 2)** | **Mean** | 2.50 | 406.50 | .01000 | .02750 | 25.50 | 63.00 | 7.00 | 5.00 |
|  |  | **SD** | .707 | 2.121 | .000000 | .024749 | 2.121 | 8.485 | .000 | .000 |
|  | **Total**  **(N= 6)** | **Mean** | 2.50 | 404.17 | .01050 | .02250 | 24.17 | 59.00 | 8.00 | 5.67 |
|  |  | **SD** | .548 | 4.622 | .000837 | .019440 | 1.602 | 6.663 | 2.000 | 1.211 |

**Table S2: Concentration of the Main Indoor Air Parameters in The Selected Office Buildings in the Study Area II**

**Office Location: II**

**Season: Wet/ Rainy Season**

**Inspection Date: May – August, 2023**

| **Cardinal point Location** | **Office Layout Type** | | **Rainy Season Average Carbon(II)Oxide (CO) Concentration (ppm)** | **Rainy Season Average Carbon(IV)Oxide (CO2) Concentration (ppm)** | **Rainy Season Average Formaldehyde (HCHO) Concentration (mg/m3)** | **Rainy Season Average Total Volatile Organic Compound (TVOC) Concentration (mg/m3)** | **Rainy Season Average Office Indoor Temperature (0C)** | **Rainy Season Average Relative Humidity (RH) (%)** | **Rainy Season Average Particulate Matter 2.5Microns (PM2.5) Concentration (ug/m3)** | **Rainy Season Office Air Quality Index (AQI)** |
| --- | --- | --- | --- | --- | --- | --- | --- | --- | --- | --- |
| **North** | **Private**  **(N= 2)** | **Mean** | 2.50 | 412.00 | .03450 | .05250 | 26.50 | 69.50 | 4.50 | 7.50 |
|  |  | **SD** | .707 | 9.899 | .002121 | .031820 | .707 | .707 | .707 | .707 |
|  | **Open Plan (N= 2)** | **Mean** | 2.50 | 420.50 | .02250 | .03750 | 26.00 | 70.50 | 7.00 | 6.00 |
|  |  | **SD** | .707 | 27.577 | .009192 | .033234 | .000 | .707 | 1.414 | 1.414 |
|  | **Cubicle /Traditional (N= 2)** | **Mean** | 2.50 | 409.00 | .02450 | .04950 | 26.00 | 69.00 | 7.00 | 6.00 |
|  |  | **SD** | .707 | 2.828 | .007778 | .040305 | .000 | 1.414 | 1.414 | 1.414 |
|  | **Total**  **(N= 6)** | **Mean** | 2.50 | 413.83 | .02717 | .04650 | 26.17 | 69.67 | 6.17 | 6.50 |
|  |  | **SD** | .548 | 14.204 | .007935 | .028261 | .408 | 1.033 | 1.602 | 1.225 |
| **East** | **Private**  **(N= 2)** | **Mean** | 3.00 | 394.50 | .02600 | .01400 | 26.00 | 68.50 | 5.50 | 5.50 |
|  |  | **SD** | .000 | 4.950 | .000000 | .001414 | .000 | .707 | 2.121 | .707 |
|  | **Open Plan (N= 2)** | **Mean** | 3.00 | 398.00 | .01700 | .01250 | 26.00 | 69.00 | 8.00 | 5.50 |
|  |  | **SD** | .000 | 4.243 | .002828 | .002121 | .000 | .000 | .000 | .707 |
|  | **Cubicle /Traditional (N= 2)** | **Mean** | 2.50 | 400.00 | .01800 | .01550 | 26.00 | 69.00 | 8.50 | 5.50 |
|  |  | **SD** | .707 | 1.414 | .004243 | .000707 | .000 | 1.414 | .707 | .707 |
|  | **Total**  **(N= 6)** | **Mean** | 2.83 | 397.50 | .02033 | .01400 | 26.00 | 68.83 | 7.33 | 5.50 |
|  |  | **SD** | .408 | 3.886 | .004967 | .001789 | .000 | .753 | 1.751 | .548 |
| **West** | **Private**  **(N= 1)** | **Mean** | 3.00 | 399.00 | .03900 | .01900 | 26.00 | 69.00 | 4.00 | 6.00 |
|  |  | **SD** | .000 | .000 | .000 | .000 | .000 | .000 | .000 | .000 |
|  | **Open Plan (N= 2)** | **Mean** | 3.00 | 401.00 | .02600 | .01500 | 25.50 | 66.00 | 3.50 | 5.50 |
|  |  | **SD** | .000 | .000 | .009899 | .005657 | .707 | 4.243 | .707 | .707 |
|  | **Cubicle /Traditional (N= 2)** | **Mean** | 2.50 | 400.50 | .02300 | .01500 | 26.00 | 69.00 | 4.50 | 4.50 |
|  |  | **SD** | .707 | 2.121 | .004243 | .005657 | .000 | .000 | .707 | .707 |
|  | **Total**  **(N= 5)** | **Mean** | 2.80 | 400.40 | .02740 | .01580 | 25.80 | 67.80 | 4.00 | 5.20 |
|  |  | **SD** | .447 | 1.342 | .008562 | .004382 | .447 | 2.683 | .707 | .837 |
| **South** | **Private**  **(N= 2)** | **Mean** | 2.50 | 403.00 | .03100 | .00600 | 25.50 | 67.00 | 5.00 | 5.00 |
|  |  | **SD** | .707 | 1.414 | .004243 | .001414 | .707 | 2.828 | 2.828 | .000 |
|  | **Open Plan (N= 2)** | **Mean** | 2.50 | 400.50 | .02700 | .00900 | 25.50 | 69.50 | 5.50 | 5.50 |
|  |  | **SD** | .707 | .707 | .001414 | .005657 | .707 | .707 | 2.121 | .707 |
|  | **Cubicle /Traditional (N= 2)** | **Mean** | 3.00 | 402.50 | .02800 | .00600 | 26.00 | 69.00 | 5.50 | 5.50 |
|  |  | **SD** | .000 | .707 | .001414 | .000000 | .000 | .000 | 2.121 | .707 |
|  | **Total**  **(N= 6)** | **Mean** | 2.67 | 402.00 | .02867 | .00700 | 25.67 | 68.50 | 5.33 | 5.33 |
|  |  | **SD** | .516 | 1.414 | .002805 | .003033 | .516 | 1.761 | 1.862 | .516 |

**Office Location: II**

**Season: Dry Season**

**Inspection Date: November – December, 2023**

| **Cardinal point Location** | **Office Layout Type** | | **Dry Season Average Carbon(II)Oxide (CO) Concentration (ppm)** | **Dry Season Average Carbon(IV)Oxide (CO2) Concentration (ppm)** | **Dry Season Average Formaldehyde (HCHO) Concentration (mg/m3)** | **Dry Season Average Total Volatile Organic Compound (TVOC) Concentration (mg/m3)** | **Dry Season Average Office Indoor Temperature (0C)** | **Dry Season Average Relative Humidity (RH) (%)** | **Dry Season Average PM2.5Microns (PM2.5) Concentration (ug/m3)** | **Dry Season Office Air Quality Index (AQI)** |
| --- | --- | --- | --- | --- | --- | --- | --- | --- | --- | --- |
| **North** | **Private**  **(N= 2)** | **Mean** | 2.00 | 398.50 | .01000 | .00550 | 27.50 | 63.00 | 13.00 | 9.00 |
|  |  | **SD** | .000 | 2.121 | .000000 | .000707 | .707 | 1.414 | .000 | .000 |
|  | **Open Plan**  **(N= 2)** | **Mean** | 2.00 | 400.00 | .01000 | .00600 | 28.50 | 63.00 | 13.50 | 9.50 |
|  |  | **SD** | .000 | .000 | .000000 | .000000 | .707 | 1.414 | .707 | .707 |
|  | **Cubicle /Traditional (N= 2)** | **Mean** | 2.00 | 400.50 | .01000 | .00550 | 28.50 | 63.50 | 13.50 | 9.50 |
|  |  | **SD** | .000 | .707 | .000000 | .000707 | .707 | 2.121 | .707 | .707 |
|  | **Total**  **(N= 6)** | **Mean** | 2.00 | 399.67 | .01000 | .00567 | 28.17 | 63.17 | 13.33 | 9.33 |
|  |  | **SD** | .000 | 1.366 | .000000 | .000516 | .753 | 1.329 | .516 | .516 |
| **East** | **Private**  **(N= 2)** | **Mean** | 3.00 | 400.00 | .01550 | .00650 | 26.00 | 63.00 | 16.00 | 11.00 |
|  |  | **SD** | .000 | 1.414 | .007778 | .000707 | .000 | 2.828 | .000 | .000 |
|  | **Open Plan**  **(N= 2)** | **Mean** | 3.00 | 399.00 | .01500 | .01000 | 27.00 | 66.50 | 16.00 | 11.00 |
|  |  | **SD** | .000 | 1.414 | .007071 | .007071 | .000 | 2.121 | .000 | .000 |
|  | **Cubicle /Traditional (N= 2)** | **Mean** | 3.00 | 400.50 | .01450 | .01100 | 27.00 | 66.50 | 15.50 | 10.50 |
|  |  | **SD** | .000 | .707 | .006364 | .008485 | .000 | 2.121 | .707 | .707 |
|  | **Total**  **(N= 6)** | **Mean** | 3.00 | 399.83 | .01500 | .00917 | 26.67 | 65.33 | 15.83 | 10.83 |
|  |  | **SD** | .000 | 1.169 | .005514 | .005382 | .516 | 2.582 | .408 | .408 |
| **West** | **Private**  **(N= 1)** | **Mean** | 3.00 | 407.00 | .01000 | .01700 | 28.00 | 65.00 | 12.00 | 8.00 |
|  |  | **SD** | .000 | .000 | .000 | .000 | .000 | .000 | .000 | .000 |
|  | **Open Plan**  **(N= 2)** | **Mean** | 3.00 | 401.50 | .01000 | .01050 | 27.50 | 62.00 | 14.00 | 9.50 |
|  |  | **SD** | .000 | 2.121 | .000000 | .007778 | .707 | 2.828 | 1.414 | .707 |
|  | **Cubicle /Traditional (N= 2)** | **Mean** | 3.00 | 401.00 | .01000 | .01100 | 27.50 | 62.50 | 14.00 | 9.50 |
|  |  | **SD** | .000 | .000 | .000000 | .007071 | .707 | 3.536 | 1.414 | .707 |
|  | **Total**  **(N= 5)** | **Mean** | 3.00 | 402.40 | .01000 | .01200 | 27.60 | 62.80 | 13.60 | 9.20 |
|  |  | **SD** | .000 | 2.793 | .000000 | .005958 | .548 | 2.588 | 1.342 | .837 |
| **South** | **Private**  **(N= 2)** | **Mean** | 3.00 | 399.50 | .01250 | .01250 | 27.00 | 61.00 | 12.00 | 8.00 |
|  |  | **SD** | .000 | .707 | .003536 | .010607 | .000 | 1.414 | 1.414 | 1.414 |
|  | **Open Plan**  **(N= 2)** | **Mean** | 2.50 | 402.00 | .01250 | .02400 | 26.00 | 59.50 | 11.50 | 7.50 |
|  |  | **SD** | .707 | 4.243 | .003536 | .026870 | .000 | .707 | .707 | .707 |
|  | **Cubicle /Traditional (N= 2)** | **Mean** | 2.50 | 405.00 | .01200 | .00550 | 26.00 | 61.50 | 11.50 | 8.00 |
|  |  | **SD** | .707 | 7.071 | .002828 | .000707 | .000 | .707 | 2.121 | 1.414 |
|  | **Total**  **(N= 6)** | **Mean** | 2.67 | 402.17 | .01233 | .01400 | 26.33 | 60.67 | 11.67 | 7.83 |
|  |  | **SD** | .516 | 4.446 | .002582 | .015388 | .516 | 1.211 | 1.211 | .983 |

**Table S3: Concentration of the Main Indoor Air Parameters in The Selected Office Buildings in the Study Area III**

**Office Location: III**

**Season: Wet/ Rainy Season**

**Inspection Date: May – August, 2023**

| **Cardinal point Location** | **Office Layout Type** | | **Rainy Season Average Carbon(II)Oxide (CO) Concentration (ppm)** | **Rainy Season Average Carbon(IV)Oxide (CO2) Concentration (ppm)** | **Rainy Season Average Formaldehyde (HCHO) Concentration (mg/m3)** | **Rainy Season Average Total Volatile Organic Compound (TVOC) Concentration (mg/m3)** | **Rainy Season Average Office Indoor Temperature (0C)** | **Rainy Season Average Relative Humidity (RH) (%)** | **Rainy Season Average Particulate Matter 2.5Microns (PM2.5) Concentration (ug/m3)** | **Rainy Season Office Air Quality Index (AQI)** |
| --- | --- | --- | --- | --- | --- | --- | --- | --- | --- | --- |
| **North** | **Private**  **(N= 2)** | **Mean** | 3.00 | 417.00 | .02050 | .01550 | 26.00 | 67.00 | 4.50 | 5.00 |
|  |  | **SD** | .000 | 22.627 | .000707 | .004950 | .000 | 1.414 | 3.536 | 1.414 |
|  | **Open Plan (N= 2)** | **Mean** | 3.00 | 418.50 | .02200 | .01550 | 26.00 | 66.50 | 4.50 | 4.50 |
|  |  | **SD** | .000 | 24.749 | .005657 | .004950 | .000 | 2.121 | 2.121 | 2.121 |
|  | **Cubicle /Traditional (N= 1)** | **Mean** | 3.00 | 401.00 | .01800 | .01200 | 26.00 | 69.00 | 3.00 | 3.00 |
|  |  | **SD** | .000 | .000 | .000 | .000 | .000 | .000 | .000 | .000 |
|  | **Total**  **(N= 5)** | **Mean** | 3.00 | 414.40 | .02060 | .01480 | 26.00 | 67.20 | 4.20 | 4.40 |
|  |  | **SD** | .000 | 18.379 | .003286 | .003834 | .000 | 1.643 | 2.168 | 1.517 |
| **South** | **Private**  **(N= 2)** | **Mean** | 3.00 | 403.00 | .02200 | .02250 | 26.00 | 67.50 | 3.50 | 4.50 |
|  |  | **SD** | .000 | 1.414 | .005657 | .019092 | .000 | 2.121 | .707 | .707 |
|  | **Open Plan (N= 2)** | **Mean** | 3.00 | 429.00 | .02200 | .02450 | 26.00 | 67.50 | 3.50 | 3.50 |
|  |  | **SD** | .000 | 38.184 | .011314 | .021920 | .000 | 2.121 | .707 | .707 |
|  | **Cubicle /Traditional (N= 1)** | **Mean** | 3.00 | 401.00 | .01500 | .01100 | 26.00 | 69.00 | 6.00 | 6.00 |
|  |  | **SD** | .000 | .000 | .000 | .000 | .000 | .000 | .000 | .000 |
|  | **Total**  **(N= 5)** | **Mean** | 3.00 | 413.00 | .02060 | .02100 | 26.00 | 67.80 | 4.00 | 4.40 |
|  |  | **SD** | .000 | 24.062 | .007057 | .015604 | .000 | 1.643 | 1.225 | 1.140 |

**Office Location: III**

**Season: Dry Season**

**Inspection Date: November – December, 2023**

| **Cardinal point Location** | **Office Layout Type** | | **Dry Season Average Carbon(II)Oxide (CO) Concentration (ppm)** | **Dry Season Average Carbon(IV)Oxide (CO2) Concentration (ppm)** | **Dry Season Average Formaldehyde (HCHO) Concentration (mg/m3)** | **Dry Season Average Total Volatile Organic Compound (TVOC) Concentration (mg/m3)** | **Dry Season Average Office Indoor Temperature (0C)** | **Dry Season Average Relative Humidity (RH) (%)** | **Dry Season Average Particulate Matter 2.5Microns (PM2.5) Concentration (ug/m3)** | **Dry Season Office Air Quality Index (AQI)** |
| --- | --- | --- | --- | --- | --- | --- | --- | --- | --- | --- |
| **North** | **Private**  **(N= 2)** | **Mean** | 3.00 | 415.00 | .01000 | .05650 | 30.00 | 57.50 | 17.00 | 11.50 |
|  |  | **SD** | .000 | 7.071 | .000000 | .014849 | .000 | .707 | 2.828 | 2.121 |
|  | **Open Plan**  **(N= 2)** | **Mean** | 3.00 | 409.50 | .01000 | .04650 | 30.50 | 58.00 | 17.00 | 12.00 |
|  |  | **SD** | .000 | .707 | .000000 | .000707 | .707 | 1.414 | .000 | .000 |
|  | **Cubicle /Traditional (N= 1)** | **Mean** | 3.00 | 410.00 | .01000 | .04800 | 29.00 | 60.00 | 16.00 | 11.00 |
|  |  | **SD** | .000 | .000 | .000 | .000 | .000 | .000 | .000 | .000 |
|  | **Total**  **(N= 5)** | **Mean** | 3.00 | 411.80 | .01000 | .05080 | 30.00 | 58.20 | 16.80 | 11.60 |
|  |  | **SD** | .000 | 4.604 | .000000 | .009094 | .707 | 1.304 | 1.483 | 1.140 |
| **South** | **Private**  **(N= 2)** | **Mean** | 3.00 | 413.00 | .01000 | .04650 | 30.00 | 56.50 | 16.50 | 11.50 |
|  |  | **SD** | .000 | 2.828 | .000000 | .000707 | .000 | .707 | .707 | .707 |
|  | **Open Plan**  **(N= 2)** | **Mean** | 2.50 | 410.00 | .01000 | .04800 | 29.50 | 57.00 | 16.50 | 11.50 |
|  |  | **SD** | .707 | .000 | .000000 | .001414 | .707 | 1.414 | .707 | .707 |
|  | **Cubicle /Traditional (N= 1)** | **Mean** | 3.00 | 412.00 | .01000 | .04600 | 29.00 | 58.00 | 16.00 | 11.00 |
|  |  | **SD** | .000 | .000 | .000 | .000 | .000 | .000 | .000 | .000 |
|  | **Total**  **(N= 5)** | **Mean** | 2.80 | 411.60 | .01000 | .04700 | 29.60 | 57.00 | 16.40 | 11.40 |
|  |  | **SD** | .447 | 2.074 | .000000 | .001225 | .548 | 1.000 | .548 | .548 |

**Table S4: ANOVA Table for raining season**

|  | **Sig**. |
| --- | --- |
| Rainy Season Average Carbon(II)Oxide (CO) Concentration * Location of Office | .233 |
| Rainy Season Average Carbon(IV)Oxide (CO2) Concentration * Location of Office | .000 |
| Rainy Season Average Formaldehyde (HCHO) Concentration * Location of Office | .030 |
| Rainy Season Average Total Volatile Organic Compound (TVOC) Concentration * Location of Office | .000 |
| Rainy season Average Office Indoor Temperature * Location of Office | .193 |
| Rainy Season Average Relative Humidity (RH) * Location of Office | .000 |
| Rainy Season Average Particulate Matter 2.5Microns (PM2.5) Concentration * Location of Office | .000 |
| Rainy Season Office Air Quality Index (AQI) * Location of Office | .000 |

**Table S5. ANOVA Table for Dry season**

|  | **Sig** |
| --- | --- |
| Dry Season Average Carbon(II)Oxide (CO) Concentration * Location of Office | .233 |
| Dry Season Average Carbon(IV)Oxide (CO2) Concentration * Location of Office | .000 |
| Dry Season Average Formaldehyde (HCHO) Concentration * Location of Office | .114 |
| Dry Season Average Total Volatile Organic Compound (TVOC) Concentration * Location of Office | .000 |
| Dry Season Average Office Indoor Temperature * Location of Office | .164 |
| Dry Season Average Relative Humidity (RH) * Location of Office | .000 |
| Dry Season Average Particulate Matter 2.5Microns (PM2.5) Concentration * Location of Office | .000 |
| Dry Season Office Air Quality Index (AQI) * Location of Office | .000 |
